# Independent effect of fat-to-muscle mass ratio at bioimpedance analysis on long-term survival in patients receiving surgery for pancreatic cancer

**DOI:** 10.3389/fnut.2023.1118616

**Published:** 2023-06-13

**Authors:** Marta Sandini, Salvatore Paiella, Marco Cereda, Marco Angrisani, Giovanni Capretti, Simone Famularo, Alessandro Giani, Linda Roccamatisi, Andrea Fontani, Giuseppe Malleo, Roberto Salvia, Franco Roviello, Alessandro Zerbi, Claudio Bassi, Luca Gianotti

**Affiliations:** ^1^Department of Medicine, Surgery and Neurosciences, Unit of General Surgery and Surgical Oncology, University of Siena, Siena, Italy; ^2^General and Pancreatic Surgery Unit, Pancreas Institute, University of Verona Hospital Trust, Verona, Italy; ^3^Department of Surgery, San Gerardo Hospital, School of Medicine and Surgery, University of Milano-Bicocca, Monza, Italy; ^4^Department of General, Hepatobiliary and Pancreatic Surgery, Liver Transplantation Service, San Camillo Forlanini Hospital, Rome, Italy; ^5^Pancreatic Surgery Unit, Department of Biomedical Sciences, Humanitas Clinical and Research Center-IRCCS Rozzano, Humanitas University, Milan, Italy; ^6^Department of Hepatobiliary and General Surgery, Humanitas Clinical and Research Center, Humanitas University, Milan, Italy

**Keywords:** bioimpedance analysis, body composition, pancreatic cancer, outcomes, surgical oncology

## Abstract

**Introduction:**

Malnutrition and alteration of body composition are early features in pancreatic cancer and appear to be predictors of advanced stages and dismal overall survival. Whether specific patient characteristics measured at the preoperative bioimpedance analysis (BIA) could be associated with long-term outcomes following curative resection has not been yet described.

**Methods:**

In a prospective multicenter study, all histologically proven resected pancreatic cancer patients were included in the analysis. BIA was measured for all patients on the day before surgery. Demographics, perioperative data, and postoperative outcomes were prospectively collected. Patients who experienced 90-day mortality were excluded from the analysis. Survival data were obtained through follow-up visits and phone interviews. Bioimpedance variables were analyzed according to the overall survival using the Kaplan–Meier curves and the univariate and multivariate Cox regression model.

**Results:**

Overall, 161 pancreatic cancer patients were included. The median age was 66 (60–74) years, and 27.3% received systemic neoadjuvant treatment. There were 23 (14.3%) patients malnourished in the preoperative evaluation. Median OS was 34.0 (25.7–42.3) months. Several bioimpedance variables were associated with OS at the univariate analysis, namely the phase angle [HR 0.85, 95% CI 0.74–0.98)], standardized phase angle [HR 0.91, 95% CI 0.82–0.99)], and an increased ratio between the fat and lean mass (FM/FFM) [HR 4.27, 95% CI 1.10–16.64)]. At the multivariate analysis, the FM/FFM ratio was a confirmed independent predictor of OS following radical resection, together with a positive lymph nodal status.

**Conclusion:**

Alteration of body composition at the preoperative bioimpedance vector analysis (BIVA) can predict dismal oncologic outcomes following pancreatic resection for cancer.

## Introduction

Pancreatic cancer (PC) has the poorest prognosis of any common solid malignancy, with a 5-year overall survival of approximately 20%. Pancreatic ductal adenocarcinoma now represents the third leading cause of overall cancer death (1), and both incidence and mortality rates increased by an average of 0.3% per year during the past decade (2). Underlying these trends is a combination of an aging population, a longer expected lifespan, and the public health pandemics of obesity and diabetes.

PC has aggressive biological characteristics. More than 50% of patients have distant metastases at presentation, and the majority of patients undergoing resection will develop local or distant recurrence within a few years after surgery, suggesting *de facto* the presence of systemic disease in patients with apparently localized tumors (3–5). The physiologic effects of PC can weaken patients, limiting their ability to withstand aggressive treatments. Some sort of nutritional derangement is present in up to 80% of PC (6). Patients with compromised nutritional status and alteration of body composition exhibit poor treatment tolerance, increased surgical morbidity, and dismal oncologic outcomes (7, 8). Preoperative alteration of different anthropometric indexes has been repeatedly associated with worsened survival after several types of major surgery (9, 10), including pancreatic resections (8). In particular, muscle mass wasting alone, or associated with obesity—the so-called sarcopenic obesity—has been reported as an independent factor for poor oncologic outcomes and increased mortality within a few years after radical pancreatic surgery (11). A systematic review and meta-analysis on this topic included 42 retrospective studies (12). Body composition assessment was carried out mainly at CT scan analysis (35 of 42), while seven studies used bioelectric impedance analysis (BIA). Even though most studies focused on patients receiving chemoradiation alone, BIA indexes were appraised to weigh the risk of short-term morbidity. To the best of our knowledge, no evidence on the association of preoperative BIA parameters in patients undergoing pancreatic surgery for PC and long-term overall survival has been provided. Despite CT remaining the reference imaging tool to estimate body compartments and their relative ratio (13), BIA has been repeatedly shown as a reliable method to assess both body composition and nutritional status (14). Therefore, we designed a prospective cohort study with the aim of assessing whether preoperative anthropometric indexes at BIA were independent predictors of long-term overall survival after pancreatic surgery for PC.

## Materials and methods

### Study overview and patient selection

Adult patients scheduled for elective pancreatic resection for PC between January 2016 and December 2018 at three Italian academic medical centers—San Gerardo Hospital, Monza, the Pancreas Institute, Verona, and Humanitas Research Hospital, Rozzano, Milan—were prospectively assessed for inclusion and asked to provide written consent. Exclusion criteria were as follows: kidney diseases with a glomerular filtration rate of < 60 mL/min and the presence of compartmentalized fluid collections (pleural effusion and peripheral edema). These conditions may interfere with the electrical properties of human tissues, resulting in unreliable body composition estimation such as fat or muscle mass. Further exclusion criteria were as follows: American Society of Anesthesiologists (ASA) score > 3; New York Heart Association > 2; presence of any infection in the previous 90 days; palliative surgery; and refusal to sign informed consent. The results are reported according to Strengthening the Reporting of Observational Studies in Epidemiology (15).

An identical electronic case report form was filled out by the three centers. Demographic data, medical history, comorbidity, malnutrition [ESPEN criteria (16)], and results from routine blood tests were collected at admission. The study protocol was approved by the ethics committees of all the institutions (Nr. 0005228).

### Bioelectrical impedance assessment

A single-frequency phase-sensitive impedance analyzer (Nutrilab^®^, Akern SRL—Pisa, Italy) was used for the BIVA. BIVA was conducted 2 h before the induction of anesthesia. The BIVA method utilizes a phase-sensitive impedance instrument that introduces a constant, low-level alternating current with a tetrapolar surface electrode placement on the hands and feet for whole-body determinations (14, 17). Impedance (Z) and the delay of current, caused by the lag of current penetrating cell membranes and tissue interfaces, are measured by low Z electrodes and expressed as phase shift or phase angle (PA). Impedance is a complex number that comprises the resistance (R) or purely resistive component (water and electrolytes in fluids and tissues) and the reactance (Xc) or capacitive component in tissues (cells and tissue interfaces). Complex electronic circuitry permits the determination of the time delay between voltage and current at the cell membrane and tissue level and thus determines the phase angle. The complex Z of an organism can be differentiated into R and Xc components with simple mathematics, Z (sin phase angle) and Z (cos phase angle), respectively, of an R–Xc series circuit for the body. Routinely, a 50-kHz phase-sensitive BIA instrument measures PA and Z and calculates R and Xc.

The standardized PA (SPA) is the observed PA—mean phase angle/standard deviation (SD), where the mean and SD are from sex-stratified, age-stratified, and BMI-stratified phase angle reference values. Hydration assessment of patients was conducted through the software Bodygram^®^ (Akern SRL—Pisa, Italy). Details of BIVA principles, measurement methods, and definitions have been previously described (18).

### Perioperative care

Pylorus-preserving pancreatoduodenectomy, classic Child operation, and distal and total pancreatectomy procedures were performed or supervised by experienced surgeons.

Perioperative care was provided *per* the Enhanced Recovery After Surgery recommendations (19). Intraoperative fluid administration was tailored to each patient according to either the variation of the cardiac output or the pulse pressure variation, through continuous radial arterial monitoring according to a goal-directed fluid therapy approach.

All postoperative complications were collected and graded according to the Clavien–Dindo classification (CDC) (20). For each complicated patient, the overall burden of postoperative morbidity was calculated *per* the comprehensive complication index (CCI) (21).

### Follow-up and long-term outcome

All patients were followed using measurement of serum carbohydrate antigen 19-9, abdominal ultrasound, contrast computed tomography or magnetic resonance imaging, and office visits. In brief, each patient was followed up every 3 months for the first 2 years and then every 6 months or on clinical demand. OS was defined as the time interval in months from surgery to death; if alive, patient data were censored at the last available visit. Patient surveillance was closed at the end of April 2022. We used the eighth edition of the American Joint Committee on Cancer staging system for PC.

### Study endpoints

The primary endpoint was to study the potential association between preoperative parameters of body composition at BIA and overall long-term survival (OS).

### Statistical analysis

The normal distribution of continuous variables was evaluated at the Kolmogorov–Smirnov test. Data are expressed as median and interquartile range (IQR). The Mann–Whitney *U*-test was used for continuous variables. Non-random association for categorical variables was tested using Fisher's exact test.

### Survival analysis for cancer patients

The Kaplan–Meier log-rank (Mantel–Cox) test and the univariate Cox proportional hazard method were used to analyze potential differences in overall survival according to the variables at the BIVA. If death was not reported during the follow-up period, patients were censored at the last available contact date.

A Cox proportional hazard model was built to assess factors independently associated with OS. The following variables were included in the model: age, the phase angle (PA), the standardized phase angle (SPA), the ratio between the fat mass and the fat-free mass (FM/FFM), the occurrence of major complications, the comprehensive complication index (CCI), and the nodal status. As the presence of disease at the specimen margins is a major determinant of OS in PC, a subgroup analysis according to the status of resection margins at the final pathology was conducted. Hazard rates (HRs) are reported with a 95% confidence interval (CI).

For each test, a two-sided *p*-value of 0.05 was considered significant. All computations were made with the IBM Corp. Released 2021. IBM SPSS Statistics, version 28.0. Armonk, NY.

## Results

Overall, 161 patients were included and analyzed. Table 1 summarizes the perioperative characteristics of the included patients. The median age at diagnosis was 66 (IQR 60–74) years, 71 (44.1%) were female, and 44 out of 161 (27.3%) had undergone neoadjuvant treatment before the operation. The median BMI was 23.7 (IQR 21.7–26.6), and 23 (14.3%) were malnourished at the time of operation. The median PA and SPA were in the normal range according to the multicenter international series (22) with 5.3° (IQR 4.6°-5.9°) and 0.20 (IQR −0.70–1.40), respectively. Most patients underwent a proximal resection (69.6%). The median follow-up time was 27 (IQR 17–43) months. The estimated overall survival (OS) for the entire cohort was 34 (95% CI 25.7–42.3) months.

**Table 1:** Perioperative characteristics of included patients.

**Variable**	**Median (IQR) or number (%) Overall N=161**
Age	66 (60-74)
Sex F/M	71/90 (44.1/55.9)
BMI	23.7 (21.7-26.6)
Malnutrition	23 (14.3)
Albumin (preoperative) (g/L)	41.2 (38.1-42.9)
PA (degrees)	5.3 (4.6-5.9)
SPA	0.20 (-0.70-1.40)
FFM	53.2 (44.9-60.1)
FM	13.7 (9.5-18.4)
TBW	39.3 (33.7-44.7)
Neoadjuvant treatment	44 (27.3)
**Postoperative pancreatic fistula**	
- Biochemical leakage - Grade B/C fistula	5 (3.1) 14 (8.7)
Biliary fistula	3 (3.7)
Major complications	17 (10.6)
Comprehensive complication index	8.7 (0.0-20.9)
**Type of operation**	
- PD	112 (69.6)
- DP	28 (17.4)
- TP	21 (13.0)
**T**	
−0-2 - 3-4	131 (81.4) 30 (18.6)
**N**	
−0 - 1 - 2	37 (23.0) 49 (30.4) 75 (46.6)
**R**	
−0 - 1	94 (58.4) 67 (41.6)
Perineural infiltration	30 (18.6)
Lymphatic infiltration	21 (13.0)
Vascular infiltration	18 (11.2)
**AJCC 18** ^th^	
- IA	17 (10.6)
- IB	17 (10.6)
- IIA	4 (2.5)
- IIB	49 (30.4)
- III	74 (46.0)

### Univariate and multivariate analyses for overall survival

Several variables measured ad BIVA were associated with overall survival, which was significantly improved together with each unitary increase in the value of the PA (*p* = 0.023) and the SPA (*p* = 0.045). On the other side, increased values of extracellular water (ECW, *p* = 0.037), adipose tissue (fat mass – FM, *p* = 0.013), and the ratio between fat mass and fat-free mass (FM/FFM, p = 0.037) were associated with dismal survival rates (Table 2).

**Table 2:** Univariate analysis of BIVA parameters for overall survival.

**Variable**	**HR (95% CI)**	***p*-value**
PA	0.85 (0.74-0.98)	0.023
SPA	0.91 (0.82-0.99)	0.045
TBW	1.01 (0.99-1.04)	0.451
HI	1.04 (0.99-1.09)	0.121
ECW	1.05 (1.01-1.09)	0.037
FFM	1.0 (0.98-1.03)	0.676
FM	1.04 (1.01-1.06)	0.013
FM/FFM	4.27 (1.10-16.64)	0.037

We ran an age-adjusted multivariate Cox proportional regression model including those BIVA variables, which showed an association with OS at the univariate analysis, together with clinical and pathology variables generally associated with OS in resected PC, namely, the occurrence of major complications, the comprehensive complication index, and a positive lymph nodal status. As the status of resection margins at pathology remains a major predictor of long-term prognosis in PC, we analyzed separately two subgroups of patients, according to the R status (R0 vs. R+). To produce a more conservative model and minimize the number of covariates, the ECW was excluded from the multivariate analysis, as this variable can directly be calculated from the PA. As shown in Table 3, a positive nodal status and the ratio between FM/FFM remained independently associated with OS after radical resection, with HR 2.29 95% CI (1.12–4.69) and HR 24.05 95% CI (3.11–186.07), respectively.

**Table 3:** Multivariate analysis for overall survival according to the pathological status at resection margins.

	**R0 (N**=**94)**		**R1 (N**=**67)**	
**Variable**	**HR (95% CI)**	**p-value**	**HR (95% CI)**	**p-value**
Age	1.02 (0.98-1.06)	0.380	0.99 (0.95-1.04)	0.752
PA	1.24 (0.75-2.05)	0.401	0.85 (0.51-1.41)	0.520
SPA	0.93 (0.69-1.24)	0.622	0.86 (0.57-1.28)	0.447
FM/FFM	24.05 (3.11-186.07)	0.002	1.57 (0.14-17.30)	0.715
CCI	1.00 (0.99-1.02)	0.645	1.02 (0.99-1.04)	0.164
Positive N status	2.29 (1.12-4.69)	0.023	0.59 (0.24-1.45)	0.254

R, resection margin status; HR, hazard ratio; PA, phase angle; FM, fat mass; FFM, fat-free mass; CCI, comprehensive complication index.

**Figure 1:**
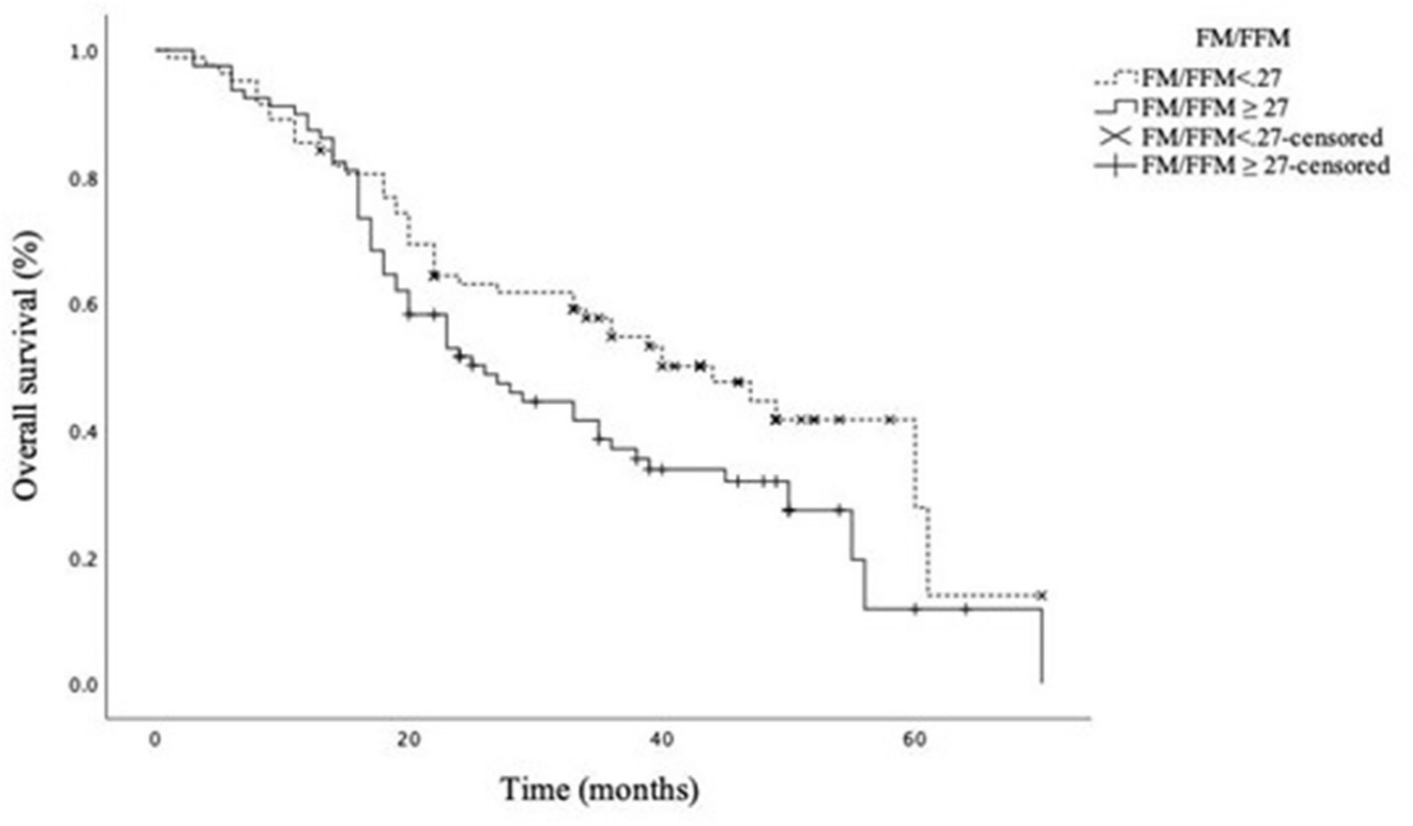
Overall survival according to the FM/FFM ratio dichotomized at the median value. Estimated median (95% CI) OS 44.0 (32.3–55.7) and 26.0 (18.7–33.3) months for FM/FFM<27 and FM/FFM ≥ 27, respectively. Log-rank (Mantel–Cox) test *p* = 0.040.

We finally modeled a Kaplan–Meier curve to compare the OS according to the ratio FM/FFM, dichotomized at the median value observed in our study cohort (FM/FFM = 27). As presented in Figure 1, a high ratio FM/FFM was associated with significantly worse OS, with an estimated median survival rate of 44 months for FM/FFM <27 versus 26 months for FM/FFM ≥ 27, p = 0.040 at log-rank (Mantel–Cox) test.

## Discussion

This prospective analysis shows that the preoperative appraisal of body composition at BIVA can be predictive of overall survival after surgical resection for pancreatic cancer. Specifically, decreased values of PA and SPA are related to unfavorable long-term prognosis following resection, while an increased ratio between FM/FFM is an independent determinant of poor overall survival (OS), with a hazard ratio (HR) of 16 (95% CI 2–139). This effect—observed in the subgroup of radical resection margins—was independent of the presence of positive lymph nodes at the final pathology.

In gastrointestinal solid cancer, the prognostic value of PA and its z-score SPA has been broadly demonstrated (23). Bioelectrical impedance is a non-invasive method of measuring body composition through the delivery of a low-frequency alternating current and works on the principle that different cellular structures have different levels of resistance to the passage of the current. The provided measures of resistance and reactance are representative of tissue hydration and cellular integrity, respectively (24). The arctangent between these latter—the phase angle—is a useful indicator of cellular health, and its clinical applications range from the evaluation of hydration status up to the stratification of long-term prognosis in oncologic settings (25). High values of PA reflect high cellularity, cell membrane integrity, and preserved cell function. In solid tumors of the head and neck and in gynecologic patients, a low PA has been associated with a more advanced stage of the disease (25). Bioimpedance data on patients suffering from pancreatic cancer are limited. Gupta et al. showed that PA with a cutoff of 5° may be predictive of survival in advanced pancreatic cancer patients (26). Nonetheless, in their study, only non-surgical advanced pancreatic cancer patients were included. Consequently, much lower PA values could have been expected in comparison with our cohort of resectable patients. Additionally, in a large study by Yasui-Yamada et al. (27) including resected gastrointestinal and hepatobiliary-pancreatic cancer patients, an association between preoperative PA and long-term outcomes was observed (27). In that cohort, the subgroup of patients suffering from PC, although resectable, showed a median PA of 4.6°, again lower than that measured in our cohort (5.3°). Higher PA values may partially explain why we observed a limited prognostic ability of PA on overall survival, which was not confirmed after adjusting for other confounders in the multivariate analysis. Despite in contrast with published data showing a high prognostic value of the PA in cancer patients, some speculative explanation can be hypothesized. Most studies analyzing the prognostic significance of PA in cancer include patients with advanced stages of the disease, who showed PA values generally lower than that in our population. The extreme observation was found in a cohort of end-stage disease admitted to an acute palliative care unit, where a PA value of lower than 3° had an accuracy of 86% for 3-day survival (28). The PA is a comprehensive parameter for assessing cellular health and function. We can postulate that such general deterioration may represent a final event in the natural history of cancer and, consequently, was not yet detectable at the time of measurement in our resectable patients.

However, in our study other parameters of body composition at BIVA were associated with long-term oncologic outcomes. An increased fat mass was predictive of reduced OS, and furthermore, the combination of high FM together with low muscularity—intended as reduced fat-free mass (FFM)—was associated with a more than 4-fold increased risk of death. This effect was also confirmed in the multivariate analysis, where a high ratio between FM/FFM was predictive of a 24-fold increase in death, following radical resection for PC. The presence of positive nodal status was also an independent predictor of OS, with an HR of 2.3 (95% QI 1.1–4.7). Muscular and adipose compartment deviations in predicting survival in both advanced and resected pancreatic cancer have been widely described. In a retrospective study including 301 resectable PC patients, Okumura et al. observed an association between visceral adiposity, sarcopenic visceral obesity, low muscle mass index, muscle attenuation, and overall survival (29). Gruber et al. observed that the preoperative presence of sarcopenia and sarcopenic obesity correlated with shorter OS, following resection for PC (30). In addition, in advanced PC, changes in body composition during the receipt of neoadjuvant treatment were associated with the likelihood of resection after neoadjuvant CT (31). The depletion of the muscular compartment alone and even more in combination with a high amount of visceral adiposity has been associated with impaired survival following resection for advanced PC patients undergoing chemoradiation, and the presence of cachectic weight loss thawed the effect of resection on OS (32). Accordingly, non-resected advanced PC showing a high visceral to subcutaneous adipose tissue ratio and low skeletal muscle index at diagnosis experienced unfavorable OS (33).

To the best of our knowledge, this is the first study to find an association between body composition and overall survival in resected PC patients by BIVA. Indeed, all published literature showed a correlation between radiologic features and prognosis. Even though a contrast-enhanced CT scan is required for clinical staging and restaging in PC, clinical aftermaths of body composition assessment at CT scan can be limited by the invasiveness of the examination. Moreover, dedicated software to process the images and interpretation from a trained radiologist are required. BIVA is a non-invasive, inexpensive, easy-to-use bedside technique and does not need any specific training to assess the body compartments. Certain body conditions provoking extreme hyperhydration or dehydration may bias the assessment of muscle mass (28); however, the use of BIVA for the determination of body compartments has been extensively validated in many healthy populations and several diseases (22, 27, 34), and the clinical feasibility of BIVA in the pancreatic surgical setting has already been confirmed in a previous trial from our research group (18).

It is well known that subclinical changes in body compartments are early manifestations of pancreatic cancer, which can occur even months before confirming the diagnosis (35). In a murine model of PC, Danai et al. observed an early activation of genes involved in autophagy and ubiquitin–proteasome degradation, suggesting the promotion of proteolysis and muscle volume depletion. In the clinical setting, a recent meta-analysis including 33 studies and more than 5,000 resectable and borderline resectable PC patients showed that the pooled prevalence of sarcopenia at diagnosis reached almost 40% (36).

Finally, the relatively small sample size and heterogeneity of our cohort in terms of pathology stage may justify why we observed the effect of BIVA on OS exclusively in the subgroup of radical resection margins. It has been broadly shown that the presence of a positive resection margin is one of the strongest determinants of OS following surgery in localized PC (37, 38). One more limitation of our study is that despite being prospective, it was not hypothesis-driven. Hence, further studies are needed to confirm the present findings. Moreover, despite widely used and validated, the accuracy of BIVA can be hampered by some medical conditions, such as severe edema, compartmentalized fluid collections, and renal failure. Even though surgical oncologic patients are not supposed to experience those conditions, this element could represent a limitation to the application of BIVA in a subgroup of patients. Finally, the measurement of FM and FFM is derived from the impedance and reactance and is not directly measured (39). As these latter can vary according to the type of impedance analyzer, the cut point of our study needs further validation according to the machine used.

## Conclusion

In conclusion, the preoperative evaluation of body composition at BIVA and in particular, the combination between increased FM and reduced FFM helps stratify post-resection long-term outcomes in localized pancreatic cancer. The technical and cost feasibility of BIVA in comparison with the CT scan should promote the implementation of BIVA in clinical practice to improve the estimation of oncologic prognosis in patients undergoing pancreatic surgery for cancer. Further studies are needed to define specific cutoffs for groups at risk of dismal post-resection survival.

## Data availability statement

The raw data supporting the conclusions of this article will be made available by the authors, without undue reservation.

## Ethics statement

The studies involving human participants were reviewed and approved by the Ethics Committee of IRCCS San Matteo Hospital, Pavia, Italy. Protocol number Nr. 0005228. The patients/participants provided their written informed consent to participate in this study.

## Author contributions

Study conception and design: MS, SP, and LG. Data collection and drafted manuscript preparation: MC, MA, AG, SF, LR, and AF. Analysis and interpretation of results: MS, GC, GM, RS, FR, AZ, CB, and LG. All authors reviewed the results and approved the final version of the manuscript.
